# Taurine Alleviates *Streptococcus uberis-*Induced Inflammation by Activating Autophagy in Mammary Epithelial Cells

**DOI:** 10.3389/fimmu.2021.631113

**Published:** 2021-03-12

**Authors:** Zhenglei Wang, Riguo Lan, Yuanyuan Xu, Jiakun Zuo, Xiangan Han, Vanhnaseng Phouthapane, Zhenhua Luo, Jinfeng Miao

**Affiliations:** ^1^Ministry of Education Joint International Research Laboratory of Animal Health and Food Safety, College of Veterinary Medicine, Nanjing Agricultural University, Nanjing, China; ^2^Shanghai Veterinary Research Institute, Chinese Academy of Agricultural Sciences, Shanghai, China; ^3^Biotechnology and Ecology Institute, Ministry of Science and Technology (MOST), Vientiane, Laos; ^4^School of Water, Energy and Environment, Cranfield University, Cranfield, United Kingdom

**Keywords:** bovine mastitis, *Streptococcus uberis*, taurine, autophagy, PTEN/Akt/mTOR signaling pathway

## Abstract

*Streptococcus uberis* infection can cause serious inflammation and damage to mammary epithelial cells and tissues that can be significantly alleviated by taurine. Autophagy plays an important role in regulating immunity and clearing invasive pathogens and may be regulated by taurine. However, the relationships between taurine, autophagy, and *S. uberis* infection remain unclear. Herein, we demonstrate that taurine augments PTEN activity and inhibits Akt/mTOR signaling, which decreases phosphorylation of ULK1 and ATG13 by mTOR and activates autophagy. Activating autophagy accelerates the degradation of intracellular *S. uberis*, reduces intracellular bacterial load, inhibits over-activation of the NF-κB pathway, and alleviates the inflammation and damage caused by *S. uberis* infection. This study increases our understanding of the mechanism through which taurine regulates autophagy and is the first to demonstrate the role of autophagy in *S. uberis* infected MAC-T cells. Our study also provides a theoretical basis for employing nutritional elements (taurine) to regulate innate immunity and control *S. uberis* infection. It also provides theoretical support for the development of prophylactic strategies for this important pathogen.

## Introduction

More than 150 different pathogens can cause mammary gland infection. Bacteria are the most common cause of mastitis (1). Antibiotics and vaccination are the primary therapeutic and prevention strategies for bovine mastitis; both can partially reduce the incidence of mammary infection and improve milk production and quality. Unfortunately, the development of a variety of drug resistance bacterial mechanisms along with the plasticity of pathogens can produce drug-resistant strains and diversified antigens enabling pathogens to resist drugs and camouflage them from the immune system (2–5). These mechanisms are especially common for intracellular pathogens. Therefore, developing effective methods to prevent and treat bovine mastitis is an urgent need.

Our recent studies have found that increasing local immunity reduces the risk of infection that is critical in preventing bovine mastitis. Mammary epithelial cells (MECs) are a major component of the mammary gland and are responsible for milk production. Although they are not bona fide immune cells, MECs have immune functions (1, 6). MECs are abundant, and their combined efforts appear to be important in host defense. *Streptococcus uberis* is an important pathogen that causes environmental bovine mastitis through complex pathogenic mechanisms that result in inflammation and damage to MECs and associated tissues (7). Previous studies in our lab have demonstrated that MECs may internalize *S. uberis*, thus empowering the bacteria to avoid elimination by drugs and host, responses. This results in severe inflammation and damage to mammary tissue (7, 8). Taurine, the most abundant free amino acid in most animal tissues, plays an important role in regulating immunity (9). It can alleviate inflammatory injury, inhibit NF-κB signaling following *S. uberis* infection, and decrease the number of intracellular bacteria (1, 10–13). The underlying mechanism by which this occurs is unknown.

Autophagy is a highly conserved self-digestion process that plays a crucial role in maintaining cellular homeostasis in response to nutrient depletion or other stresses, such as accumulation of damaged organelles, unneeded protein aggregation, and invading microbes (14). Autophagy may have varying effects on different pathogens and hosts. For instance, it has emerged as an innate immune response pathway that targets intracellular pathogens to restrict their replication in the cytosol (15). As an example, *Mycobacterium tuberculosis, Shigella*, and *Listeria* can be degraded by autophagy (16, 17). Conversely, the replication of some pathogens relies on autophagy in host cells. *Brucella* for example selectively co-opts autophagy initiation complexes that convert *Brucella*-containing vacuoles (BCV) into a compartment with autophagic features (aBCV) to subvert host clearance and facilitate bacterial replication in the endoplasmic reticulum, promoting infection (18). These findings indicate that autophagy is a “double-edged sword” that is advantageous or harmful to the host depending on the pathogen, host species, and other conditions. There are no reports on the role of autophagy in *S. uberis* infected MECs. It has been reported that various pathways participate in the regulation of autophagy, such as RAF1 (RAF proto-oncogene serine/threonine-protein kinase)/MEK1/2 (dual specificity mitogen-activated protein kinase 1,2)/ERK1/2 (mitogen-activated protein kinase), PI3K (phosphatidylinositol 3-kinase)/Akt (protein kinase B)/mTOR (mammalian target of rapamycin), and AMPK (adenosine 5′-monophosphate (AMP)-activated protein kinase)/ULK1 (unc-51-like kinase 1) (19, 20). In particular, the PI3K/Akt/mTOR pathway plays an important role in regulating autophagy and has emerged as a critical pathway in coordinating inflammatory responses (21–24). Additionally, the PI3K/Akt/mTOR pathway is closely related to the phosphoinositides (PIs) (25, 26). Previous research in our laboratory have demonstrated that *S. uberis* induces inflammatory responses in EpH_4_-Ev cells mainly through TLR2, but the role of TLR4 cannot be ignored. Inhibition of PI3K/Akt/mTOR signaling can alleviate *S. uberis*-induced inflammation in MECs (7). Besides, Taurine plays an important role in regulating PIs/Ca^2+^ signaling (10). However, the precise relationship between taurine, TLRs, and mTOR signaling is unclear.

Based on published reports and preliminary research from our laboratory, we hypothesize that taurine regulates autophagy through mTOR signaling and autophagy plays an important role in host anti-*S. uberis* infection. In this study, the bovine mammary epithelial cell line MAC-T, *S. uberis* 0140J, and taurine were used to investigate the mechanism(s) of taurine regulation of autophagy in alleviation of *S. uberis* induced injury in MECs. The results of this study provide novel ideas and theoretical support for the prevention and control of mastitis.

## Materials and Methods

### Bacterial Strains, Cell Lines, Bacteria and Cell Culture, and Treatment

The bovine mammary epithelial cell line MAC-T was a generous gift of Dr. Loor (University of Illinois at Urbana-Champaign, Champaign, IL, USA). *S. uberis* 0140 J was purchased from the American Type Culture Collection (Manassas, VA, USA). Bacteria were inoculated into Todd-Hewitt broth (THB) supplemented with 2% fetal bovine serum (FBS; Gibco, USA) in an orbital shaker and grown to mid-log phase (OD_600_ of 0.5). MAC-T cells were incubated in DMEM with 10 % FBS and plated at 80 % confluence into 6-well-plates, and then treated according to the different test conditions.

### Total Protein Extraction and Western Blot

Cells were washed twice in 2 mL ice-cold PBS and harvested with a rubber policeman after being lysed on ice for 20 min in lysis buffer (Beyotime, Shanghai, China). Equal amounts of total protein were solubilized by sodium dodecyl sulfate (SDS) sample buffer (Beyotime, Shanghai, China), separated by SDS-polyacrylamide gel electrophoresis (SDS-PAGE), and transferred to a polyvinylidene difluoride membrane (Millipore, Bedford, MA, USA). Membranes were incubated with corresponding polyclonal and secondary antibodies. Signal was detected with a Super ECL Reagent substrate (Hai Gene, Harbin, China). Source and product number of antibody: LC3B (Abcam, ab48394), SQSTM1/p62 (Abcam, ab101266), Beclin 1 (Bioworld, AP0769), p-ULK1 (Affinity Biosciences, AF4387), ULK1 (Proteintech, 20986-1-AP), p-ATG13 (Bioworld, BZ40743), ATG13 (Bioworld, bs6045), ATG5 (Proteintech, 10181-2-AP), p-PI3K (CST, 4228), PI3K (CST, 4249), p-PTEN (CST, 9554), PTEN (CST, 9188), p-Akt (CST, 13038), Akt (CST, 4691), p-mTOR (Abcam, ab84400), mTOR (CST, 2972S), p-p70 S6K (CST, 9205), p70 S6K (CST, 2708), p-4E-BP1 (CST, 2855), 4E-BP1 (CST, 9644), GAPDH (Bioworld, AP0066), HRP-linked antibody (anti-rabbit IgG, CST, 7074), Goat Anti-Rabbit (Alexa Fluor®488) (Abcam, ab150077), goat anti-rabbit (Alexa Fluor®647) (Abcam, ab150079).

### TNF-α, IL-1β, IL-6, PIP_2_, and PIP_3_ ELISA

TNF-α, IL-1β, and IL-6 in MAC-T cells were measured by ELISA kits according to the manufacturer's instructions (Rigor Bioscience, Beijing, China). PIP_2_, IP_3_ in cells were detected by commercial ELISA kits (Jianglaibio, Shanghai, China). Briefly, prepared standards and enzyme labeled antibodies were reacted for 60 min at 37°C. Plates were washed five times. Chromogen solutions A and B were added and incubated for 10 min at 37°C. Stop solution was added, and OD measured at 450 nm within 10 min. TNF-α, IL-1β, and IL-6 were expressed as ng/g of protein and PIP_2_, IP_3_ were expressed as ng/mg of protein. Qualitative differences or similarities between the control and experimental groups were consistent throughout the study.

### Transfection and Inhibitor Treatment of MAC-T Cells

Cells were transfected with 10 ng/μL mCherry-EGFP-LC3 plasmid for 24 h using Lipofectamine 3000 reagent (Thermo, USA) or 20 nmol·L^−1^ siRNA (siTauT, siPAT1, or siATG5) for 48 h using riboFECTTM CP (RiboBio, Guangzhou, China) according to the manufacturers' instructions. siTauT and siPAT1 processing was according to procedures established by our lab (10). The sequences of siRNA were designed and listed as follows: siTauT (SLC6A6): GGATAGCCAGTTTGTGGAA; siPAT1 (SLC36A1): CCAATGGGACCACCAACAA; and siATG5: GATATGGTTTGAATATGAA. Inhibitors treatment: 50 nmol·L^−1^ Bafilomycin A1 (Baf-A1, inhibitor of H^+^-ATPase, Sellect Chemicals, USA) for 1 h; 2 μmol·L^−1^ VOTH (VO-Ohpic trihydrate, inhibitor of PTEN, Sellect Chemicals, USA) for 24 h; 5 mmol·L^−1^ 3-Methyladenine (3-MA, inhibitor of autophagy, Sellect Chemicals, USA) for 24 h; 100 nmol·L^−1^ Rapamycin (Rapa, inhibitor of mTOR, Sellect Chemicals, USA) for 2 h.

### Immunofluorescence

Cells were washed with PBS and fixed for 15 min in 4% paraformaldehyde. Cells were then permeabilized with 0.5% PBST for 20 min and then blocked in PBS containing 5% goat serum (Bioworld, Nanjing, China) for 2 h, followed by incubation with primary antibodies diluted in goat serum-PBS overnight. The cells were washed with PBS and incubated with secondary antibodies diluted in goat serum-PBS for 2 h. The cells were counterstained with DAPI (Solarbio, Beijing, China) for 10 min and washed 5 times in PBS (5 min each). The cells were then sealed with anti-fluorescence quenching agent and observed under a laser scanning confocal microscope. All steps were performed at room temperature.

### Transmission Electron Microscopy

Cells were treated and harvested by trypsinization, washed three times with PBS, fixed with a buffer containing 2.5% glutaraldehyde for 24 h, and then refixed in 1% osmium tetroxide for 2 h. The cells were then dehydrated in a graded ethanol series, washed with propylene oxide, and embedded in embedding medium. The samples were sectioned on a ultramicrotome at 90 nm thickness. The ultrathin sections were stained with uranyl acetate and lead citrate. Images were obtained using a transmission electron microscope (FEI T12, FEI, USA) at 80 kv.

### Viable Bacteria Count

Viable bacteria were enumerated as colony-forming units (CFU) on THB agar medium. Cells were washed with PBS and added to 100 μg/mL gentamicin to kill extracellular bacteria. Harvested cells were lysed in sterile tri-distilled water and the supernatant was collected by centrifugation at 1,000 rpm for 10 min. The CFUs of the supernatant was counted by the plate count method after incubation for 12 h at 37°C.

### Statistical Analysis

All data are expressed as means ± standard error of the mean (SEM). Experiments were performed in triplicate. Statistical procedures were computed using SPSS17.0 statistical software (SPSS Inc., Chicago, IL, USA), and *P*-values for significance were calculated with one-way analysis. Statistically significant results are indicated with ^*^ or ^#^(*P* < 0.05).

## Results

### Taurine Activates Autophagy in MAC-T Cells by Promoting LC3B-II Processing and Autophagosome Formation

**Figure 1:**
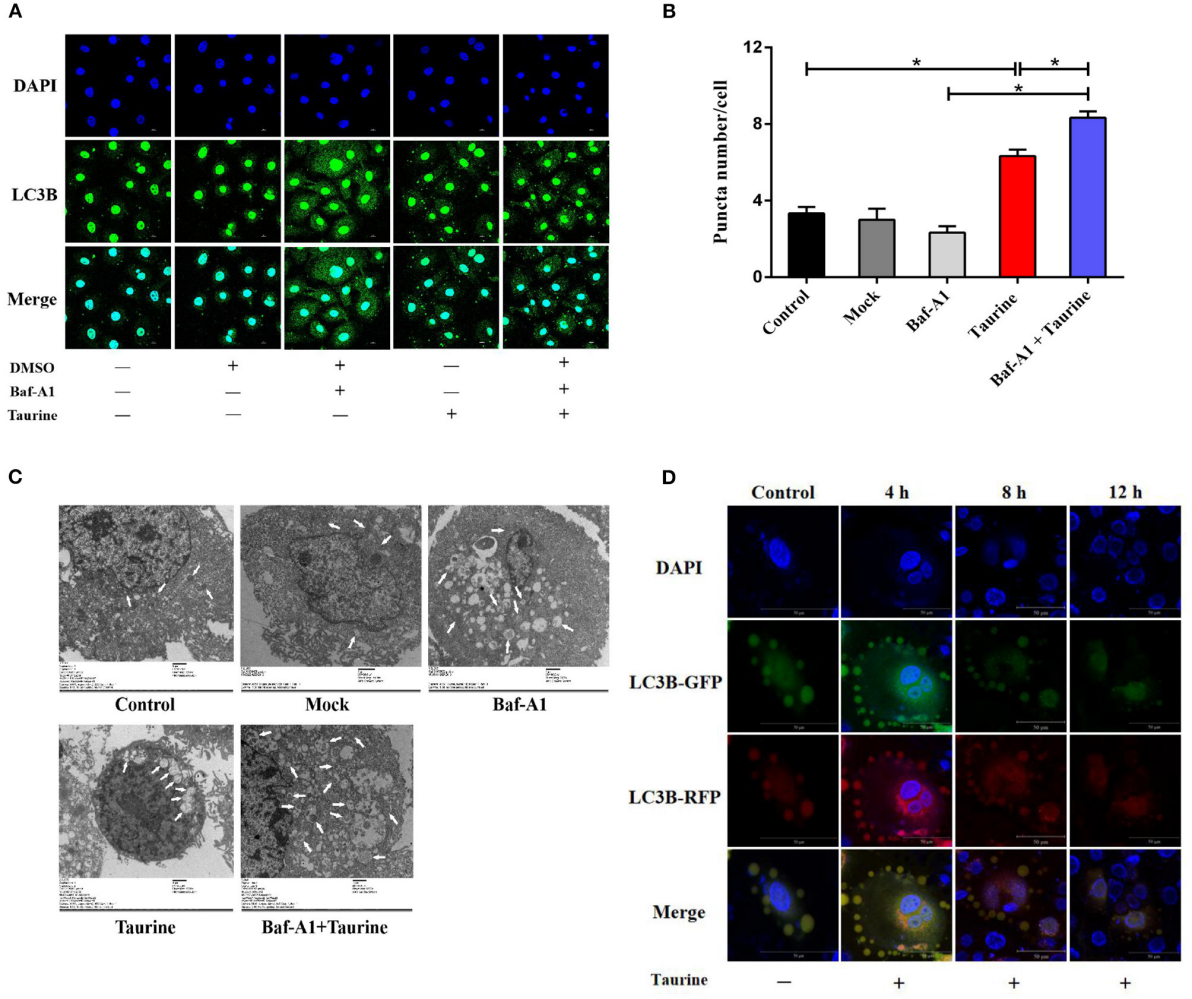
Taurine-induced autophagy in MAC-T cells. **(A,B)** Representative confocal images of LC3B puncta (green) and DAPI (blue) in MAC-T cells incubated with 50 nmol·L^−1^ Baf-A1 for 1 h before treatment with 70 mmol·L^−1^ taurine for 4 h. **(C)** TEM images of autophagosomes in MAC-T cells incubated with 50 nmol·L^−1^ Baf-A 1 for 1 h before treatment with 70 mmol·L^−1^ taurine for 4 h. White arrows indicate autophagosomes. **(D)** Fluorescence images of MAC-T cells expressing mCherry-EGFP-LC3 untreated (control) or treated with taurine. Green: GFP; Red: RFP(mCherry); Yellow: merge. Scale bar: 50 μm. Data are presented as the mean ± SEM. *P < 0.05 (significantly different) between the indicated groups.

To examine whether taurine regulates autophagy, MAC-T cells were incubated with 50 nmol·L^−1^ bafilomycin A1 (Baf-A1), a lysosome inhibitor that blocks degradation of both autophagosomes and LC3B-II, for 1 h before treatment with different concentrations of taurine. LC3B- II and p62 (sequestosome 1) levels were determined, and Beclin 1 proteins assayed (Supplementary Figures 1A,B). Treatment with 50–90 mmol·L^−1^ taurine significantly increased LC3B-II, p62, and Beclin 1 protein levels; 70 mmol·L^−1^ taurine had the most significant effects (*P* < 0.05). Taurine further increased LC3B-II, p62, and Beclin 1 accumulation in cells treated with Baf-A1. These results suggest that taurine does not inhibit autophagosome degradation, but instead induces autophagic flux since additional increases of LC3B-II result from treatment with lysosome inhibitor reflecting the amount of newly produced autophagosomes. To determine the optimal timing of taurine regulated autophagy, MAC-T cells were treated with 70 mmol·L^−1^ taurine for 0.5–24 h (Supplementary Figures 1C,D). The results demonstrate that taurine activates the classic autophagic response characterized by the accumulation of LC3B-II. Treatment for 2–8 h had significant effects, with the maximal effect occurring at 4 h (*P* < 0.05). Immunostaining of LC3B in cells exposed to 70 mmol·L^−1^ taurine for 4 h have an accumulation of cells with LC3B puncta, whereas control cells have reduced and diffuse staining (Figures 1A,B). Transmission electron microscopy on MAC-T cells treated with taurine confirmed that taurine triggers autophagosome formation. As shown in Figure 1C, greater autophagosome formation is present in treated vs. control cells. To analyze autophagic flux in greater detail, we generated cells expressing mCherry-EGFP-LC3 which allows for discrimination between early autophagosomes with dual red and green fluorescence, and autolysosomes with only red fluorescence (27). Compared with untreated cells, taurine-treated MAC-T cells have more autophagosomes that gradually changed from yellow to red, indicating an increased number of activated autolysosomes (Figure 1D). These results indicate that taurine increases autophagosome formation and lysosomal activity, resulting in the induction of autophagic flux in MECs. MECs treated with 70 mmol·L^−1^ taurine for 4 h activates autophagy in MAC-T cells. This concentration and duration was used for all further experiments.

### Taurine Activates Autophagy in an mTOR-Dependent Manner

**Figure 2:**
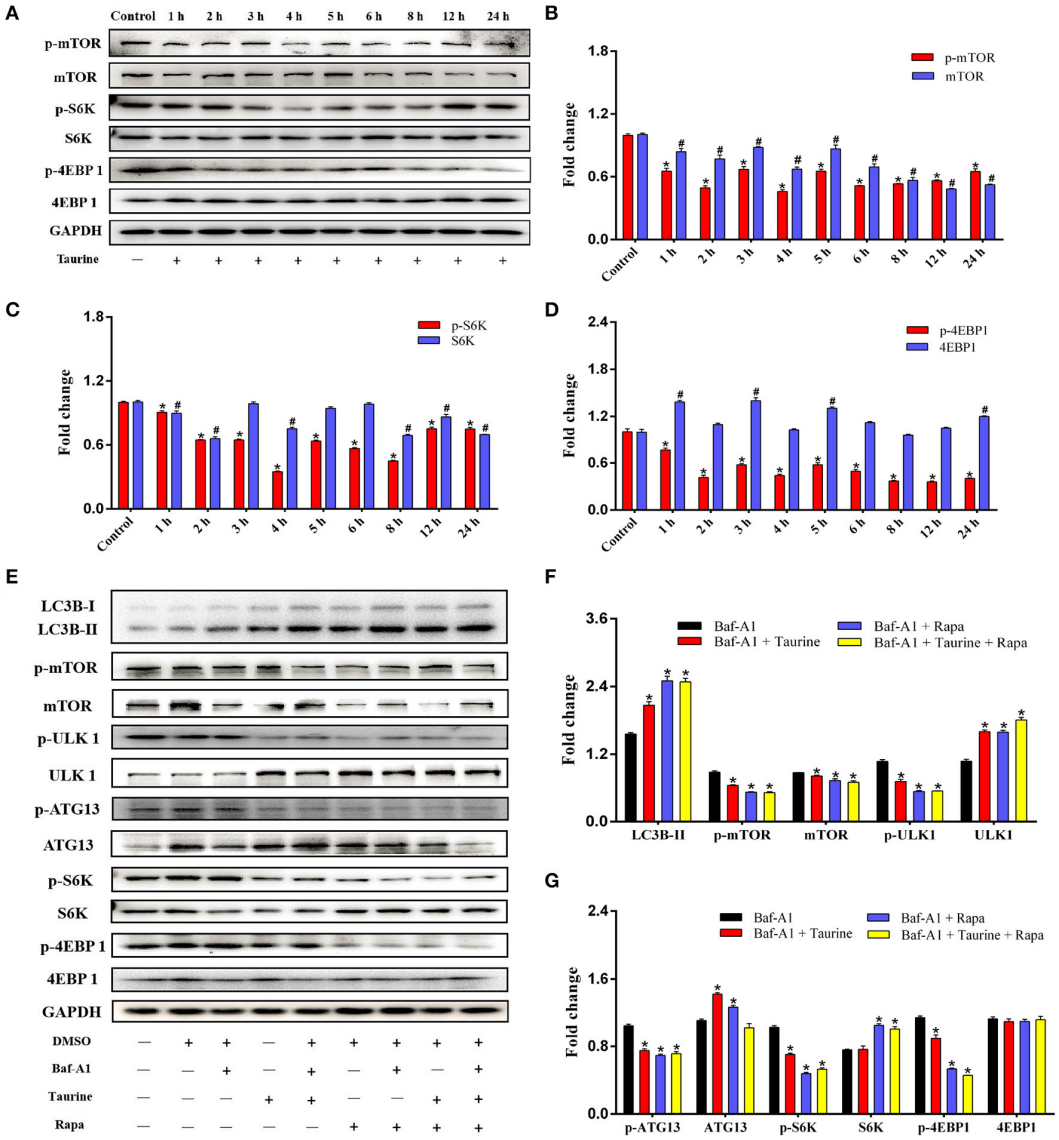
Taurine treatment decreases mTOR activity and inhibits phosphorylation of ULK1 and ATG13 in MAC-T cells. **(A–D)** Immunoblots of total protein from MAC-T cells treated with 70 mmol·L^−1^ taurine for different durations. Quantification of the ratio between the total and phosphorylated (p–) proteins, as determined by densitometric scanning of immunoblots (*n* = 3). Data are presented as mean ± SEM. **(E–G)**. Cells were incubated with 50 nmol·L^−1^ Baf-A1 for 1 h, and then treated with 70 mmol·L^−1^ taurine for 4 h or 100 nmol·L^−1^ Rapa for 2 h or both; all cells were then probed for the total and phosphorylated (p–) proteins shown. Data are presented as mean ± SEM. *P < 0.05 (significantly different) between the indicated groups; ^#^*P* < 0.05 (significantly different) between the corresponding negative control groups.

mTOR activity was analyzed in cells treated with taurine for 1–24 h. The results demonstrate that phosphorylation of mTOR and its major downstream effectors, ribosomal protein S6 kinase (S6K) and eukaryotic initiation factor 4E-binding protein 1 (4EBP1), are significantly decreased (*P* < 0.05; Figures 2A–D). These data indicate that taurine regulates autophagy in a mTOR-dependent manner. ULK1 and autophagy-related protein 13 (ATG13) are required for the initiation and formation of autophagosomes. They are phosphorylated by mTORC1 and inhibit the ability of ULK1 and ATG13 to initiate autophagy (28). To investigate whether taurine regulates autophagy through this mechanism, we used rapamycin (Rapa), a specific mTOR inhibitor, as a positive control to investigate the influence of taurine on ULK1 and ATG13 (Figures 2E–G and Supplementary Figures 2A,B). Cells treated with taurine or Rapa, or both, have significantly increased LC3B-II levels and decreased phosphorylation of mTOR, ULK1, ATG13, S6K, and 4EBP1 (*P* < 0.05). These results indicate that taurine has similar effects as Rapa on regulating autophagy. Taurine may inhibit mTOR signaling and decrease phosphorylation of ULK1 and ATG13 initiating autophagy in MAC-T cells.

### Taurine Inhibits the PI3K/Akt/mTOR Signaling Pathway Inducing Autophagy

**Figure 3:**
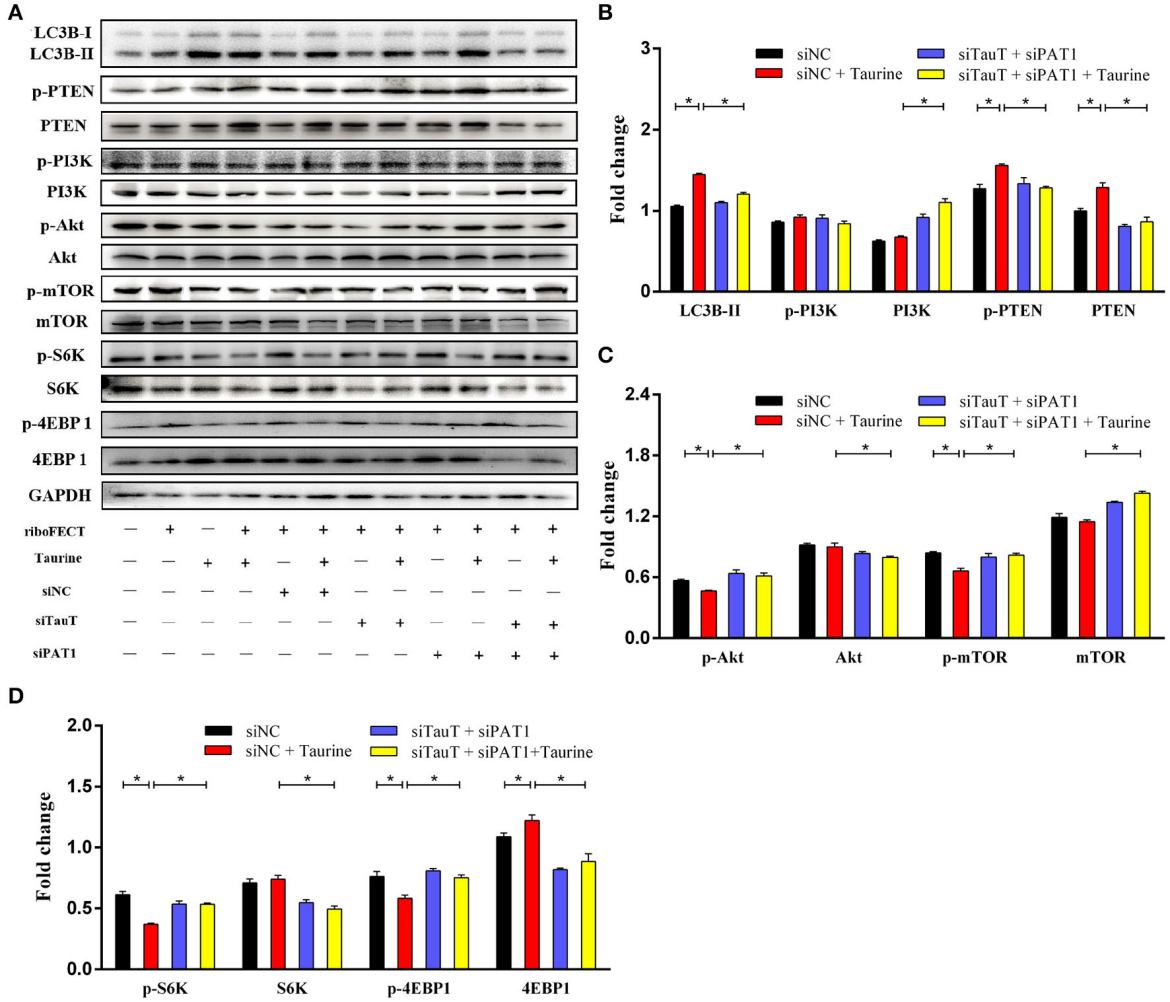
Taurine inhibits the mTOR signaling pathway. **(A–D)** Representative immunoblots of total protein from MAC-T cells transfected with 20 nmol·L^−1^ siNC, siTauT, siPAT1, or both siTauT and siPAT1 for 48 h before treatment with 70 mmol·L^−1^ taurine for 4 h. Quantification of the ratio between total and phosphorylated (p–) proteins, as determined by densitometric scanning of immunoblots and normalization to GAPDH (*n* = 3). Data are presented as mean ± SEM. *P < 0.05 (significantly different) between the indicated groups.

**Figure 4:**
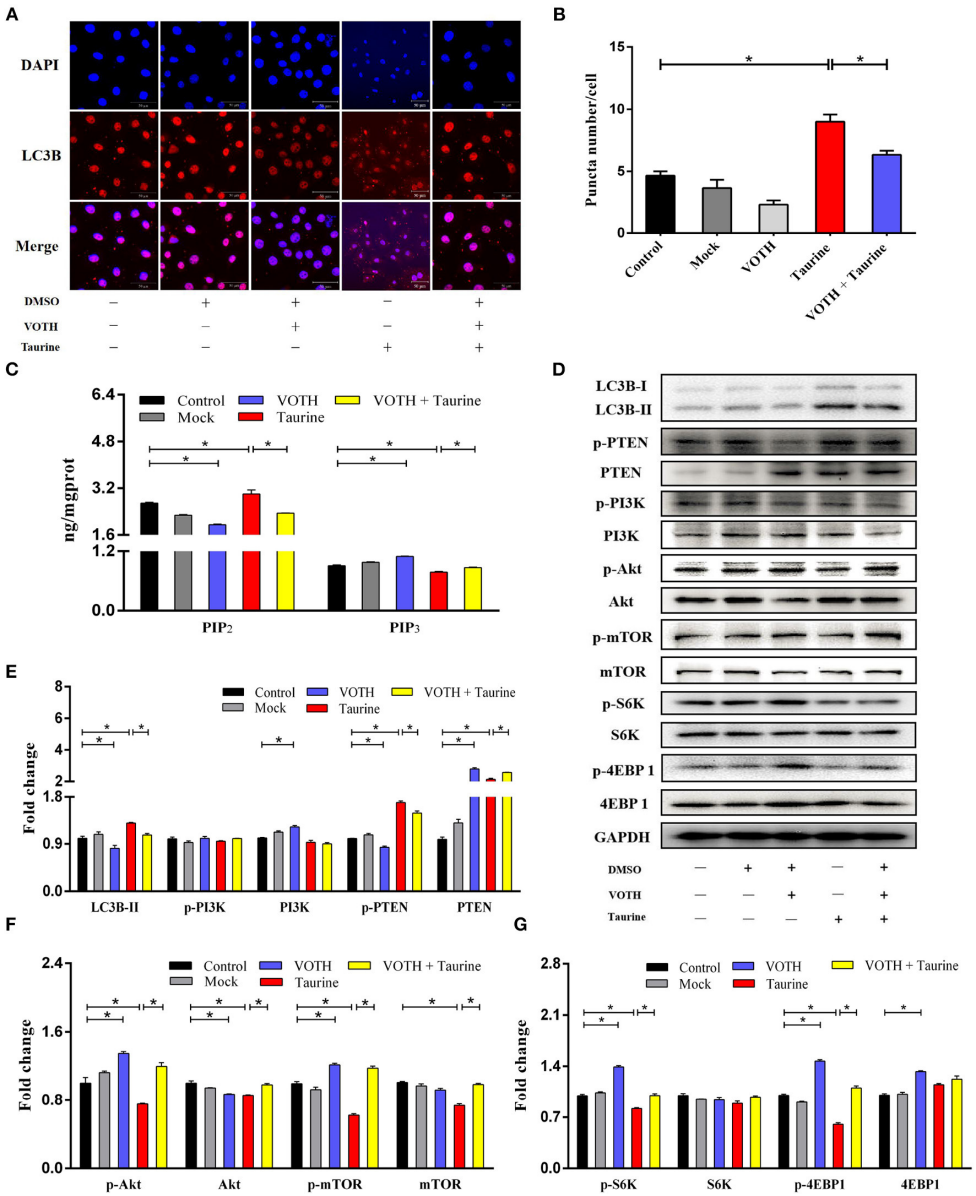
Taurine augments PTEN activity inhibited mTOR signaling and activated autophagy. **(A,B)** Confocal images of LC3B puncta (red dot) in MAC-T cells incubated with 2 μmol·L^−1^ VOTH (VO-OHpic trihydrate) for 24 h before treatment with 70 mmol·L^−1^ taurine for 4 h. **(C)** PIP_2_ and PIP_3_ concentrations were determined by ELISA in the same cells. **(D–G)** Representative immunoblots of total protein from MAC-T cells. Quantification of the ratio between total and phosphorylated (p–) proteins, as determined by densitometric scanning of immunoblots and normalization to GAPDH (*n* = 3). Data are presented as mean ± SEM. *P < 0.05 (significantly different) between the indicated groups.

Evidence suggests that Akt/mTOR signaling regulates cellular growth, metabolism, migration, differentiation, and autophagy that are predominantly linked to the lipid phosphatase activity of the tensin homolog deleted on chromosome 10 (PTEN), through which PTEN antagonizes the PI3K pathway (29, 30). To investigate the mechanism of taurine regulation of mTOR signaling, cells were transfected with siTauT and siPAT1 (*TauT* and *PAT1* encode taurine transporters). As shown in Figures 3A–D and Supplementary Figures 2C–E, cells treated with taurine have significantly increased LC3B-II, PTEN, and phosphorylated PTEN (p-PTEN) levels. Simultaneously, there are decreased Akt, mTOR, and phosphorylated levels of Akt (p-Akt) and mTOR (p-mTOR). These effects of taurine were blocked when *TauT* and *PAT1* were silenced (*P* < 0.05). There was no significant effect on PI3K activation. Thus, taurine may regulate PTEN activity inhibiting Akt/mTOR signaling and activating autophagy in MECs. PTEN is a dual-specificity phosphatase with protein- and lipid-phosphatase activities. PTEN counteracts Akt/mTOR signaling by catalyzing the conversion of phosphatidylinositol 3,4,5-trisphosphate (PIP_3_) into phosphatidylinositol 4,5-bisphosphate (PIP_2_) (30). To further investigate whether taurine regulates the mTOR pathway by modulating PTEN activity, MECs were incubated with VO-Ohpic trihydrate (VOTH), a specific inhibitor of PTEN. As shown in Figures 4A,B, taurine significantly increases LC3B puncta compared with controls, while VOTH pretreatment significantly reduces LC3B puncta (*P* < 0.05). These results indicate that taurine activates autophagy through its regulation of PTEN activity. PIP_3_ and PIP_2_ are substrates and products of PTEN and may be utilized to measure the phosphatase activity of PTEN (30). To further determine if taurine affected PTEN activity, PIP_2_ and PIP_3_ concentrations were analyzed by ELISA (Figure 4C). The results demonstrate that taurine significantly increases PIP_2_ and decreases PIP_3_, while VOTH significantly increases PIP_3_ and decreases PIP_2_. VOTH inhibits the effects of taurine (*P* < 0.05). These data indicate that taurine increases PTEN activity. To evaluate the interaction of PTEN with the PI3K/Akt/mTOR signaling pathway, protein levels of LC3B-II, PTEN, PI3K, Akt, and mTOR, as well as the phosphorylated levels of PTEN (p-PTEN), PI3K (p-PI3K), Akt (p-Akt), and mTOR (p-mTOR) were determined by Western blot (Figures 4D–G). Taurine significantly increases LC3B-II, PTEN, and phosphorylated PTEN (p-PTEN). In contrast, there were decreased levels of mTOR protein, phosphorylated Akt (p-Akt), mTOR (p-mTOR), and its downstream effectors S6K (p-S6K) and 4EBP1 (p-4EBP1) (*P* < 0.05). There was no significant effect on PI3K (*P* > 0.05). Compared with the taurine supplemented groups, pretreatment with VOTH prior to taurine treatment significantly decreases LC3B-II and phosphorylated PTEN (p-PTEN) and increases PTEN, PI3K, mTOR and phosphorylated Akt (p-Akt), mTOR (p-mTOR), S6K (p-S6K), and 4EBP1 (p-4EBP1) (*P* < 0.05). Thus, taurine increases PTEN activity, inhibits Akt/mTOR signaling and activates autophagy in MAC-T cells.

### Taurine Activated Autophagy Alleviates Inflammation and Damage Caused by *S. uberis* Infection

**Figure 5:**
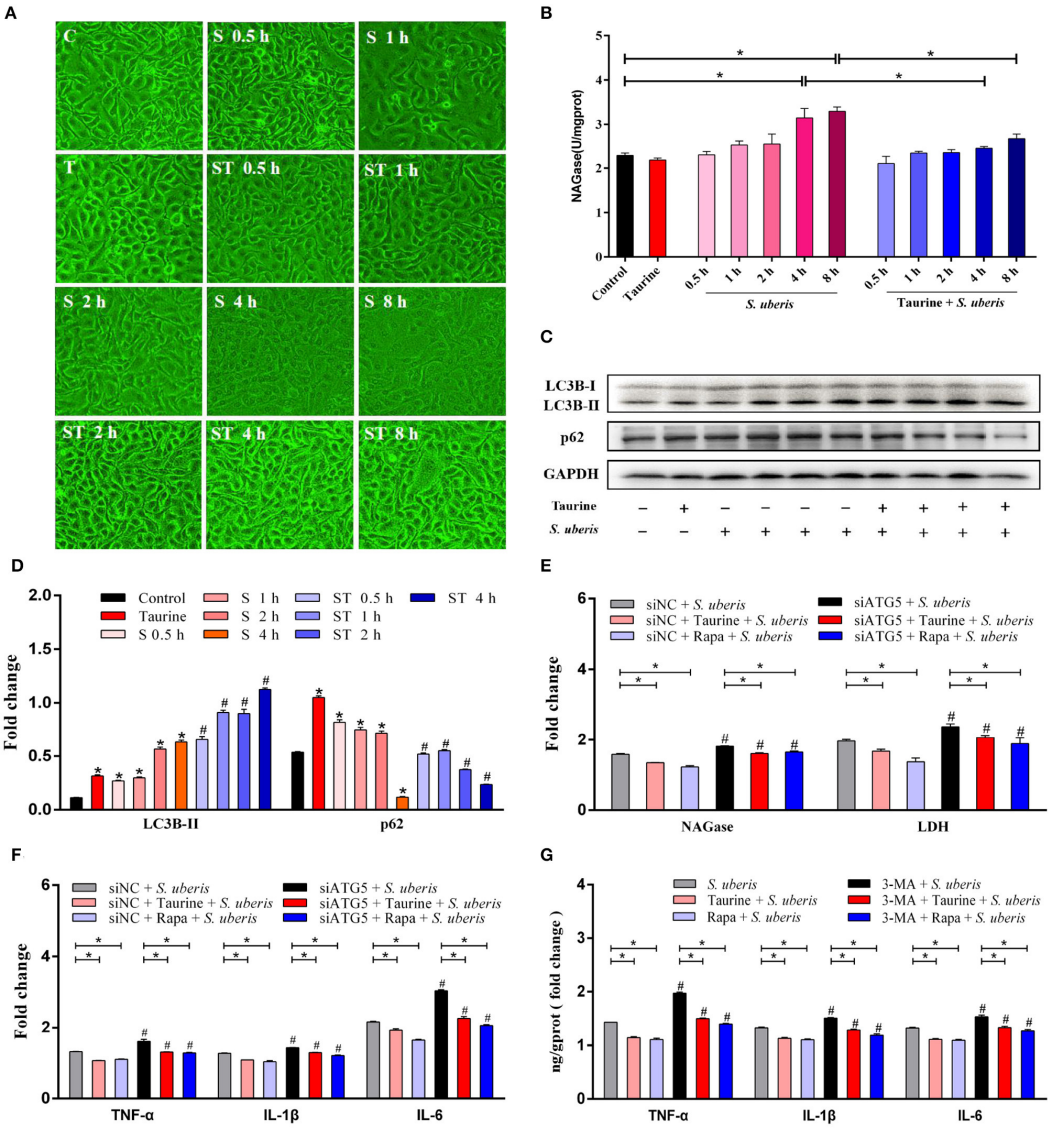
Taurine alleviates inflammation and injury caused by *S. uberis* infection. **(A)** Observations of cellular morphology by inverted microscope. MAC-T cells were treated with 70 mmol·L^−1^ taurine for 4 h, and then infected with *S. uberis* at a multiplicity of infection (MOI) of 10 for different time. Groups: C (untrated), T (treated with 70 mmol·L^−1^ taurine for 4 h), S 0.5 h (infected with *S. uberis* at MOI of 10 for 0.5 h), S 1 h (infected with *S. uberis* at MOI of 10 for 1 h), S 2 h (infected with *S. uberis* at MOI of 10 for 2 h), S 4 h (infected with *S. uberis* at MOI of 10 for 4 h), S 8 h (infected with *S. uberis* at MOI of 10 for 8 h), ST 0.5 h (treated with 70 mmol·L^−1^ taurine for 4 h, then infected with *S. uberis* at MOI of 10 for 0.5 h), ST 1 h (treated with 70 mmol·L^−1^ taurine for 4 h, then infected with *S. uberis* at MOI of 10 for 1 h), ST 2 h (treated with 70 mmol·L^−1^ taurine for 4 h, then infected with *S. uberis* at MOI of 10 for 2 h), ST 4 h (treated with 70 mmol·L^−1^ taurine for 4 h, then infected with *S. uberis* at MOI of 10 for 4 h), ST 8 h (treated with 70 mmol·L^−1^ taurine for 4 h, then infected with *S. uberis* at MOI of 10 for 8 h). **(B)** NAGase activity in supernatant was determined using commercial kits. **(C,D)** Representative immunoblots of total protein from MAC-T cells treated with 70 mmol·L^−1^ taurine for 4 h, or infected with *S. uberis* in mid-exponential phase at MOI of 10 for 0.5, 1, 2, and 4 h or both. Quantification of the ratio between the indicated proteins, as determined by densitometric scanning of immunoblots and normalization to GAPDH (*n* = 3). **(E)** NAGase and LDH activities in supernatant were determined using commercial kits. MAC-T cells transfected with 20 nmol·L^−1^ siNC or siATG5 for 48 h were treated with 70 mmol·L^−1^ taurine for 4 h or 100 nmol·L^−1^ Rapa for 2 h, and infected with *S. uberis* in mid-exponential phase at MOI of 10 for 4 h. **(F)** The concentrations of TNF-α, IL-1β, and IL-6 in supernatant were determined by ELISA. MAC-T cells transfected with 20 nmol·L^−1^ siNC or siATG5 for 48 h then treated with 70 mmol·L^−1^ taurine for 4 h or 100 nmol·L^−1^ Rapa for 2 h, and then infected with *S. uberis* in mid-exponential phase at MOI of 10 for 4 h. **(G)** The concentrations of TNF-α, IL-1β, and IL-6 in supernatant were determined by ELISA. MAC-T cells incubated with 5 mmol·L^−1^ 3-MA for 24 h were then treated with 70 mmol·L^−1^ taurine for 4 h or 100 nmol·L^−1^ Rapa for 2 h, and then infected with *S. uberis* in mid-exponential phase at MOI of 10 for 4 h. Data are presented as mean ± SEM. *P < 0.05 (significantly different) between the indicated groups; ^#^*P* < 0.05 (significantly different) between the corresponding negative control groups.

To explore the effects of taurine on *S. uberis* infection, cells pretreated with taurine were incubated with *S. uberis* for different durations. As shown in Figure 5A, cells became significantly round, shrunken, and detached after 4 and 8 h of *S. uberis* infection. These changes were markedly decreased by taurine pretreatment. NAGase (*N*-acetyl-β-D-glucosaminidase), a marker enzyme used to determine the extent of damage to MECs, was increased in cell culture supernatant 4 and 8 h post-infection (*P* < 0.05; Figure 5B). Because the cellular damage was well advanced following the 8 h challenge, *S. uberis* infection for 4 h was employed. Compared with control groups, taurine or *S. uberis* infection increased LC3B-II and p62 (*P* < 0.05). Taurine pretreatment prior to infection with *S. uberis* further improved LC3B-II and decreased p62 (*P* < 0.05; Figures 5C,D). These data demonstrate that taurine significantly alleviates damage caused by *S. uberis* infection. This phenomenon may relate to activation of autophagy by taurine in MAC-T cells.

*S. uberis*-infected MECs transfected with siRNA to silence autophagy related protein 5 (ATG5), a key regulatory gene for autophagy, or treated with Rapa (autophagy activator) or taurine were infected by *S. uberis*. As shown in Figure 5E and Supplementary Figures 3A,B, LDH (lactic dehydrogenase) and NAGase, marker enzymes used to determine the extent of damage to MECs, were increased in cell culture supernatant 4 h post-challenge (*p* < 0.05). Both taurine and Rapa pretreatment significantly decrease the activities of NAGase and LDH in the *S. uberis*-challenged groups (*P* < 0.05). The activities of NAGase and LDH in supernatant are higher in the siATG5 plus *S. uberis* groups compared to the siNC plus *S. uberis* groups (*P* < 0.05). Compared with the control groups, tumor necrosis factor (TNF-α), interleukin-1β (IL-1β), and interleukin-6 (IL-6) were also significantly increased in *S. uberis* groups, while pretreatment with taurine or Rapa significantly decreased the concentration of these cytokines (*P* < 0.05). Compared with the siNC plus *S. uberis* groups, the concentration of TNF-α, IL-1β, and IL-6 were higher in the siATG5 plus *S. uberis* groups. Silencing ATG5 lessens the ability of taurine and Rapa to decrease the concentration of these cytokines after *S. uberis* infection (*P* < 0.05; Figure 5F and Supplementary Figures 3C–E). 3-Methyladenine (3-MA) is an inhibitor of autophagy. 3-MA treatment results in similar outcomes to those of siATG5 treatment (Figure 5G and Supplementary Figure 3F). These data demonstrate that autophagy plays an important role in *S. uberis* infection, suggesting taurine and Rapa alleviate *S. uberis* inflammation and damage caused by autophagy.

**Figure 6:**
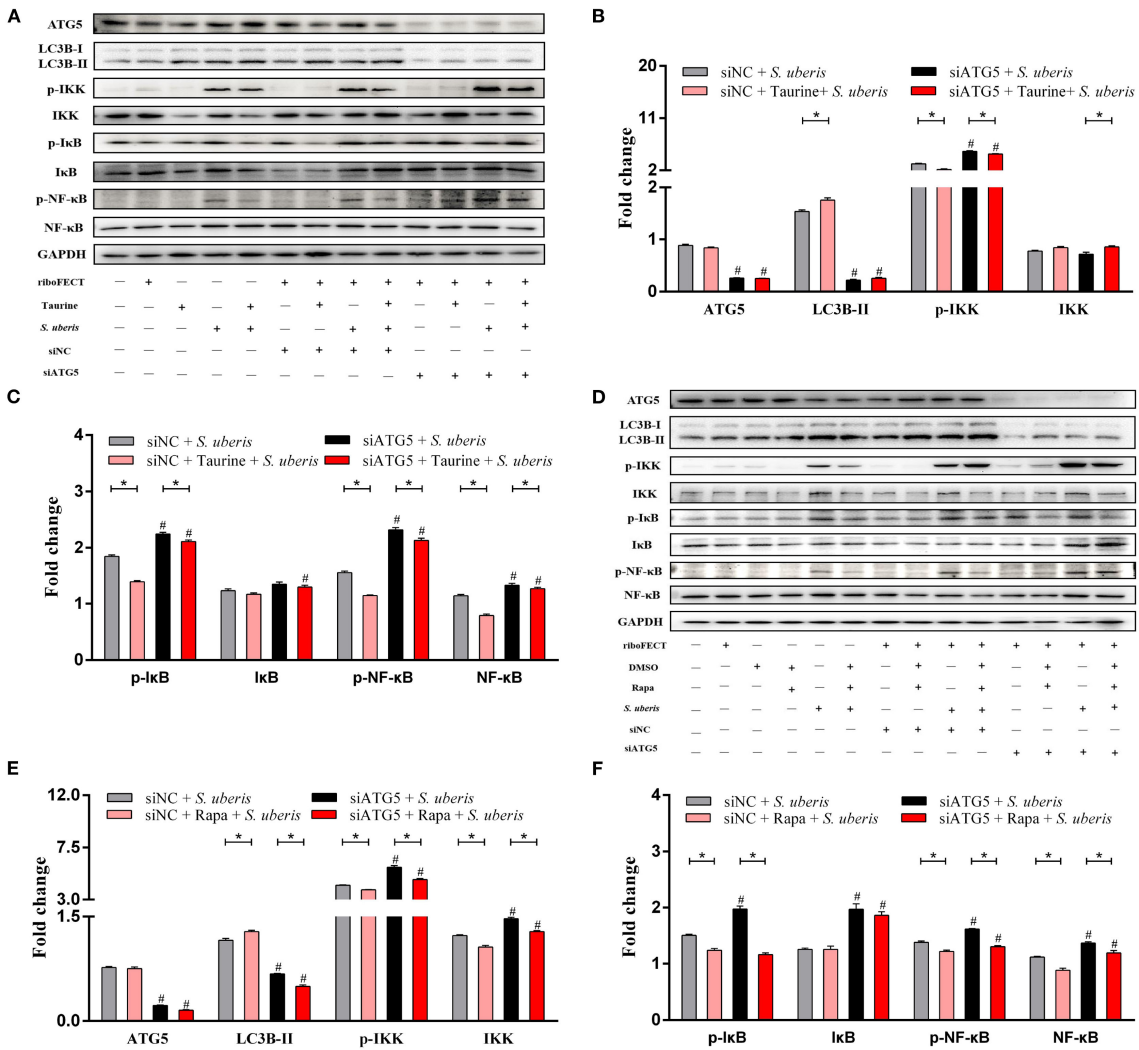
Taurine and Rapa both activate autophagy, which inhibits *S. uberis*-induced NF-κB activation. **(A–F)** Representative immunoblots of total protein from MAC-T cells transfected with 20 nmol·L^−1^ siNC or siATG5 for 48 h prior to treatment with 70 mmol·L^−1^ taurine for 4 h or 100 nmol·L^−1^ Rapa for 2 h, and then infected with *S. uberis* in mid-exponential phase at MOI of 10 for 0.5 h, 1 h, 2 h, and 4 h. Quantification of the ratio between total and phosphorylated (p–) proteins, as determined by densitometric scanning of immunoblots and normalization to GAPDH (*n* = 3). Data are presented as mean ± SEM. *P < 0.05 (significantly different) between the indicated groups; ^#^*P* < 0.05 (significantly different) between the corresponding negative control groups.

The nuclear factor kappa B (NF-κB) family of transcription factors plays an essential role in inflammation and innate immunity (31, 32). To clarify the effect of autophagy on NF-κB signaling, the protein expression and phosphorylation status of NF-κB pathway members and autophagy related proteins were determined by Western blot (Figures 6A–F and Supplementary Figures 4A–D). MAC-T cells transfected with siATG5 had significantly decreased ATG5 protein levels, significantly decreased LC3B-II and decreased autophagy (*P* < 0.05). Compared with the control groups, *S. uberis* infection significantly increases phosphorylated IKK (inhibitor of nuclear factor kappa-B kinase) (p-IKK), IκB (inhibitor of nuclear factor kappa-B kinase) (p-IκB), and NF-κB (p-NF-κB), while pretreatment with taurine or Rapa significantly decreases the phosphorylation of these proteins (*P* < 0.05). When autophagy is inhibited by siATG5 prior to *S. uberis* infection, the levels of phosphorylated IKK (p-IKK), IκB (p-IκB), and NF-κB (p-NF-κB) were further increased, decreasing the ability of taurine or Rapa to block phosphorylation of these proteins (*P* < 0.05). These data further demonstrate that autophagy plays an important role in *S. uberis* infection, and that taurine and Rapa inhibition of the overactivation of inflammatory signaling caused by *S. uberis* infection may relate to autophagy activation.

### Taurine Accelerates the Degradation of Intracellular *S. uberis* by Activating Autophagy and Reducing the Intracellular Bacterial Load

**Figure 7:**
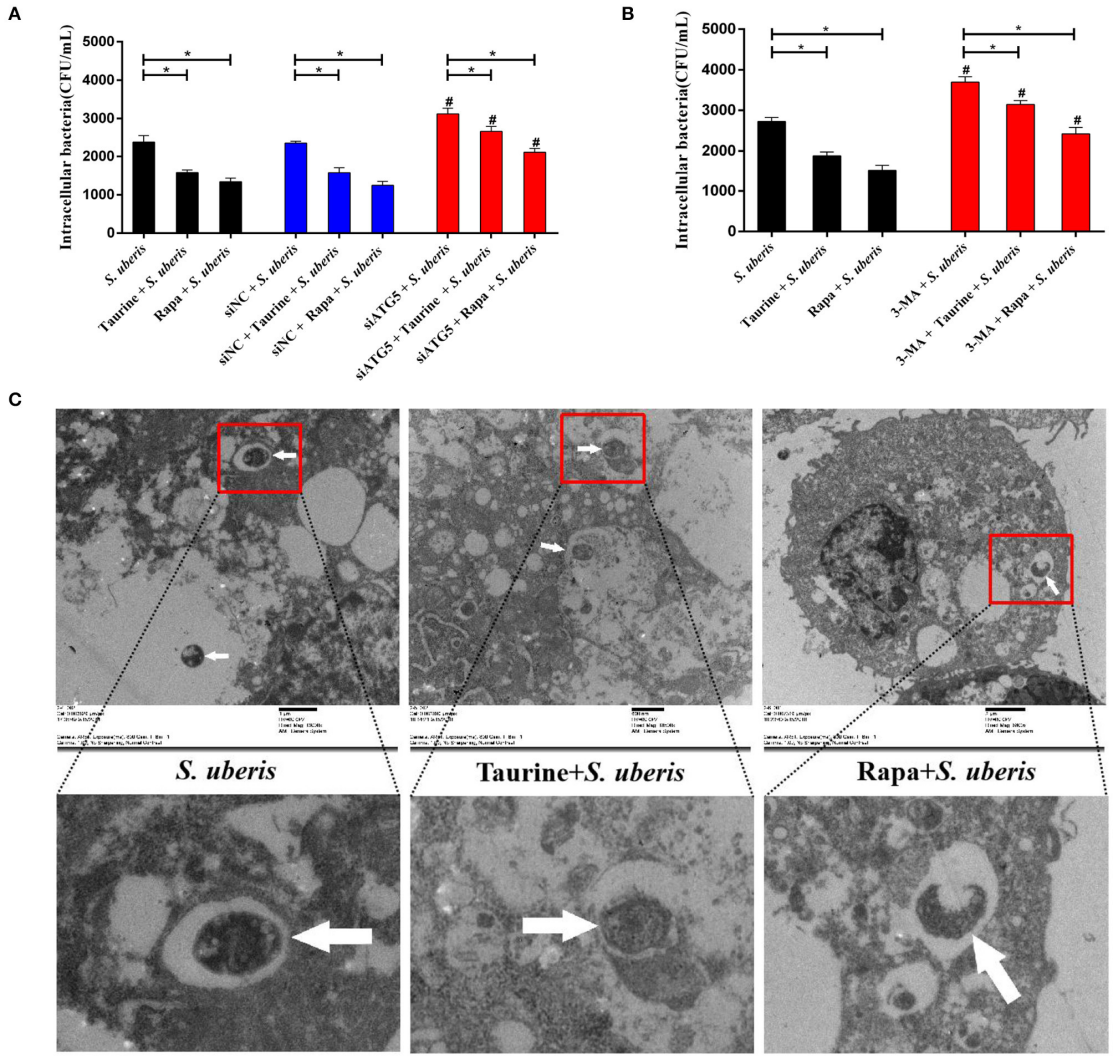
Autophagy reduces intracellular bacteria. **(A)** Cells of each treatment group were lysed with sterile tri-distilled water after washing with PBS (100 μg·mL^−1^ gentamicin). Viable bacteria were enumerated as colony-forming units (CFU) on THB agar medium. MAC-T cells transfected with 20 nmol·L^−1^ siNC or siATG5 for 48 h were then treated with 70 mmol·L^−1^ taurine for 4 h or 100 nmol·L^−1^ Rapa for 2 h, and then infected with *S. uberis* in mid-exponential phase at MOI of 10 for 4 h. **(B)** MAC-T cells incubated with 5 mmol·L^−1^ 3-MA for 24 h were then treated with 70 mmol·L^−1^ taurine for 4 h or 100 nmol·L^−1^ Rapa for 2 h, and then infected with *S. uberis* in mid-exponential phase at MOI of 10 for 4 h. Viable bacteria were enumerated as colony-forming units (CFU) on THB agar medium. **(C)** *S. uberis* were observed by electron microscopy. MAC-T cells treated with 70 mmol·L^−1^ taurine for 4 h or 100 nmol·L^−1^ Rapa for 2 h were then infected with *S. uberis* in mid-exponential phase at MOI of 10 for 4 h. White arrows indicate *S. uberis* in MAC-T cells. Data are presented as mean ± SEM. *P < 0.05 (significantly different) between the indicated groups; ^#^*P* < 0.05 (significantly different) between the corresponding negative control groups.

Autophagy is a homeostatic process that directly sequesters cytoplasmic components for degradation. It functions as a defense mechanism against invading intracellular pathogens targeting both vacuolar and cytosolic pathogens (33). To determine whether degradation of intracellular *S. uberis* is mediated by autophagy, we examined the intracellular bacterial load. As shown in Figures 7A,B, the quantity of viable bacteria was lower in the taurine or Rapa plus *S. uberis* group compared with the *S. uberis* challenge only group (*P* < 0.05). When pretreated with siATG5 or 3-MA to inhibit autophagy prior to *S. uberis* challenge, viable bacteria counts were higher compared to the *S. uberis* challenge only groups. Blocking autophagy significantly decreases the ability of taurine and Rapa to reduce intracellular bacterial load (*P* < 0.05). Ultrastructural analysis shows that *S. uberis* are viable in the *S. uberis* only groups, while pretreatment with taurine or Rapa accelerates the degradation of intracellular *S. uberis* as demonstrated by bacterial cell wall damage, blurred structure, and partial degradation (Figure 7C). These results further demonstrate that activating autophagy accelerates the degradation of intracellular *S. uberis* and reduces intracellular bacterial load.

## Discussion

Taurine is an important multifunctional amino acid reported to effect autophagy. Bai et al. (34) demonstrated that taurine protects against As_2_O_3_-induced autophagy in juvenile rat livers. Li et al. (35) showed that taurine inhibits autophagy induced by perfluorooctane sulfonates (PFOS) in PC12 cells. Other studies have shown that taurine activates autophagy. Kaneko et al. (36) demonstrated taurine activates autophagy in adipocytes. Taurine transporter deficient mice (TauT KO) exhibit decreased autophagic flux and impaired ATP-dependent 26S β5 proteasome activity in cardiac cells (37). These discrepant results may arise from different experimental conditions and cell types. In the current study, we show that taurine promotes LC3B-II, Beclin 1, autophagosome formation, autophagic flux, and other autophagy markers, demonstrating taurine activated autophagy in MAC-T cells.

One of the well-known mechanisms for autophagy is the mTOR pathway (38). mTOR is a serine/threonine protein kinase that comprises mTORC1 and mTORC2. mTORC1 is a major negative regulator of autophagy that inhibits this process by phosphorylating ULK1 and ATG13. Phosphorylation of ULK1 by mTORC1 results in suppression of its catalytic activity, which inhibits autophagy initiation (39). In a similar manner, mTORC1-dependent ATG13 phosphorylation negatively influences its activity and translocation to autophagy initiation sites (39). In the experiments described herein, we demonstrate that taurine and Rapa increase LC3B-II, and decrease phosphorylation of mTOR, ULK1, and ATG13. Thus, taurine activates autophagy in an mTOR-dependent manner in MAC-T cells. These data are consistent with previous studies. For example, Luo et al. (40) found Rapa activated autophagy via the mTORC1/ULK1/ATG13 signaling pathway in mouse aortic smooth muscle cells.

mTOR activity is regulated by a variety of upstream proteins. PTEN is a major negative regulator catalyzing PIP_3_ to PIP_2_ through its lipid phosphatase activity, inhibiting the activity of Akt and mTOR (30). PTEN has many domains with different functions; the C terminal domain contains a PDZ domain and CK2 phosphorylation sites that play an important role in regulating protein stability and phosphatase activity (30). Phosphorylation of PTEN increases its stability and inhibits its degradation or cleavage by the proteasome and caspase 3. Patsoukis et al. (41) report that casein kinase 2 (CK2) phosphorylates the C-terminal domain at Ser370, Ser380, Thr382, Thr383, and Ser385, increasing PTEN stability and reducing its activity. Phosphorylation at different sites of PTEN have different effects on its enzymatic activity. Li et al. (42) showed that RhoA-associated kinase (ROCK) phosphorylates the C2 domain at Ser229, Thr232, Thr319, and Thr321, increasing PTEN activity. Al-Khouri et al. (43, 44) found the effects on PTEN stability and activity of C-terminal domain phosphorylation at Ser385 by casein kinase 1 (CK1) or at Ser380, Thr382, Thr383, and Ser385 by liver kinase B1 (LKB1) are unclear. In the study described herein, we found that taurine promotes autophagy increased PTEN protein and phosphorylation levels, and inhibits phosphorylation of Akt and mTOR, that were decreased by inhibiting taurine-transportation (siTauT, siPAT1). We conclude that taurine regulation of Akt/mTOR signaling is related to its effect on PTEN activity. When PTEN activity is inhibited by VOTH, the ability of taurine to increase autophagosomes, catalyze PIP_3_ to PIP_2_, and inhibit Akt and mTOR activity are diminished. Thus, we infer that taurine promotes the activity of PTEN to negatively regulate Akt/mTOR signaling and activate autophagy in MAC-T cells. These results are consistent with previous studies. He et al. (45) showed that taurine exhibits an apoptosis-inducing effect on human nasopharyngeal carcinoma cells by promoting PTEN activity inhibiting Akt signaling *in vitro*. Transcriptional factor EB (TFEB) is a positive regulatory transcription factor of autophagy that is phosphorylated by mTOR, which inhibits its activity (46, 47). Kaneko et al. (36) suggested that taurine enhances TFEB nuclear translocation through ERK1/2 to activate autophagy. They reported that taurine inhibits mTOR activity, which may also reduce the inhibitory effect on TFEB by inhibiting mTOR activity and activating autophagy. In addition, It has been previously reported that the gut microbiota converts taurine to sulfide and hydrogen sulfide promotes autophagy through the PI3K/Akt/mTOR signaling pathway (48, 49). These findings support our results and indicate that taurine-induced autophagy is related to PTEN/Akt/mTOR signaling in MAC-T cells.

Numerous studies have revealed that autophagy plays an important role in infection, immunity, and clearing invasive pathogens. Zhang et al. (50) found that baicalin alleviated *Mycobacterium tuberculosis-*induced inflammation via inducing autophagy in macrophages. In the current study, taurine significantly alleviates MAC-T cell damage and morphological changes caused by *S. uberis* infection. Pretreatment with taurine prior to *S. uberis* infection further activates autophagy compared with treatment with taurine or infection by *S. uberis* alone. Thus, we postulate that autophagy may have an important role in resisting *S. uberis* infection in MECs. To test this, siATG5 or 3-MA were used to inhibit autophagy in conjunction with Rapa or taurine to activate autophagy prior to *S. uberis* infection. These experiments demonstrate that inhibiting autophagy by siATG5 or 3-MA increases NAGase activity, LDH pro-inflammatory cytokine production and activation of NF-κB signaling in response to *S. uberis* infection. These levels are reduced when activating autophagy by Rapa or taurine. Inhibiting autophagy decreases the ability of Rapa and taurine to alleviate the damage and inflammation caused by *S. uberis* infection. We thus confirm that autophagy plays a positive role in MAC-T cells infected by *S. uberis*. Furthermore, activating autophagy by Rapa or taurine accelerates the degradation of *S. uberis*, decreases the number of viable intracellular bacteria and reduces the stimulation of *S. uberis* on the immune system, which may be an underlying mechanism for how taurine alleviates the inflammation and damage caused by *S. uberis*. These data are consistent with previous studies. Nozawa et al. (51) found that Group A *Streptococcus* (GAS) was selectively sequestered within GAS-containing autophagosome-like vacuoles (GcAVs) and was killed upon the fusion of GcAVs with lysosomes. Tumbarello et al. (52) demonstrated that *Salmonella typhimurium* becomes ubiquitinated, which subsequently triggers the recruitment of selective autophagy receptors, such as SQSTM1/p62, optineurin, and NDP52, that targets bacteria and degrades them by autophagy. Consistent with these data, autophagy has an important role in alleviating the inflammation and damage caused by *S. uberis*.

**Figure 8:**
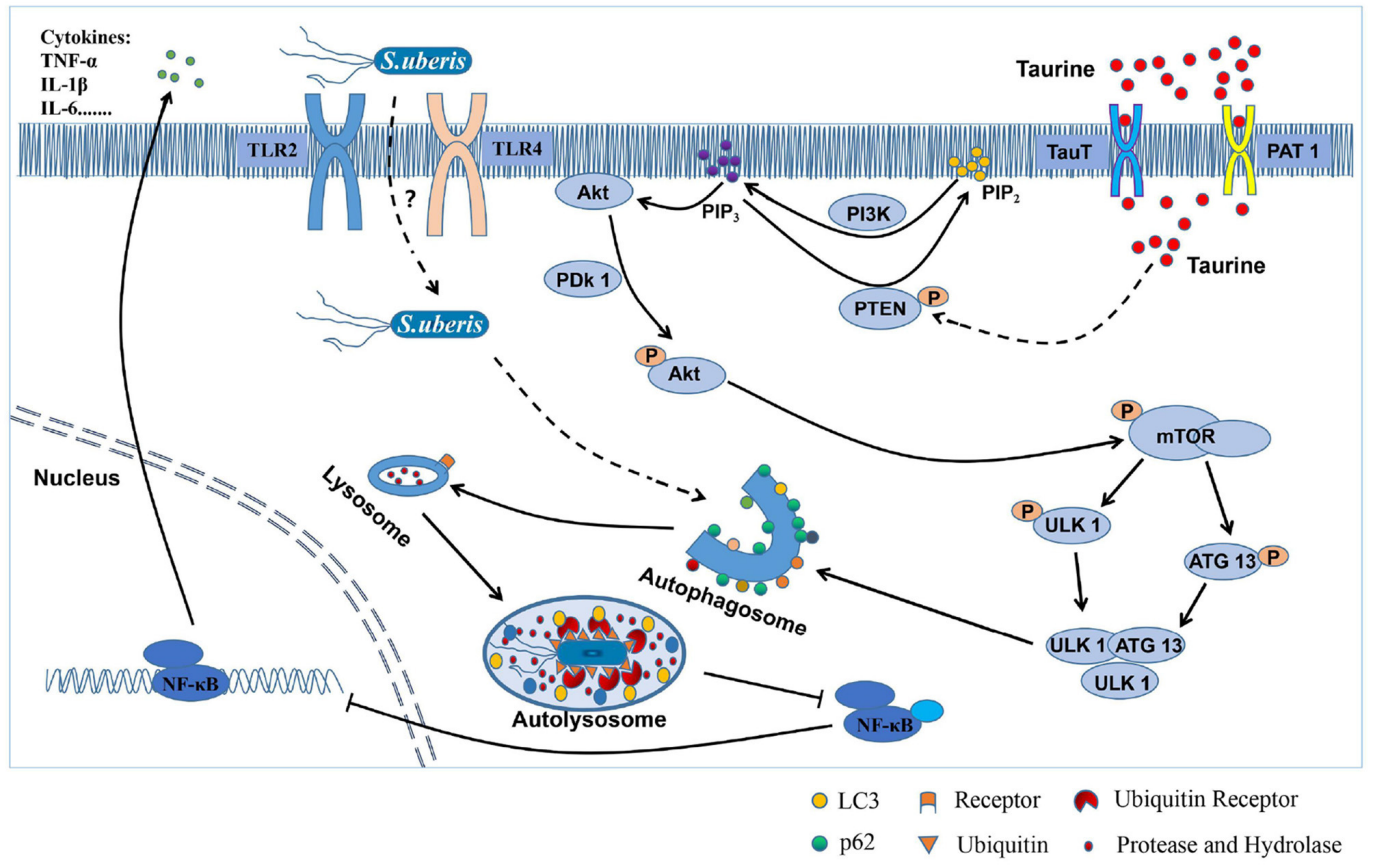
The mechanism of how taurine relieves *S. uberis* infection by activating autophagy. From the above results, we infer that taurine enters the cells through transporters (TauT and PAT 1) to improve PTEN activity, promote the conversion of PIP_3_ to PIP_2_, and inhibit PI3K/Akt/mTOR signaling pathway, thereby attenuating the phosphorylation of ULK1 and ATG13 by mTOR, promoting the formation of ULK1-ATG13-FIP200 complex, and activating autophagy of MAC-T cells. *S. uberis* is recognized by both TLR2 and TLR4, internalizes into cells and may be ubiquitinated. Then, it is mediated by related receptor proteins into autophagosomes and fused with lysosomes to form autolysates. Finally, *S. uberis* is degraded and eliminated by proteases and hydrolases, which attenuates the stimulation to cells and reduces the activation of NF-κB, inhibits the secretion of inflammatory factors (such as TNF-α, IL-1β, and IL-6), and alleviates the excessive inflammatory response and damage caused by *S. uberis* infection.

In summary, taurine inhibits mTOR signaling by activating PTEN inducing autophagy that accelerates the degradation of intracellular *S. uberis* decreasing intracellular bacterial load, inhibits over-activation of inflammatory responses, and alleviates the damage caused by *S. uberis* infection in MAC-T cells (Figure 8). This study provides a theoretical basis for using nutritional elements to regulate the body's innate immunity and improve the ability to clear *S. uberis* infection. Thus, it provides a solid basis for the development of prophylactic strategies for this important disease.

## Data Availability Statement

The original contributions presented in the study are included in the article/Supplementary Material, further inquiries can be directed to the corresponding author/s.

## Author Contributions

ZW performed the whole experiments and wrote the manuscript. RL and JZ provided assistance for data acquisition, data analysis, and statistical analysis. YX and XH participated in the design of this study. VP collected important background information. ZL performed manuscript review. JM carried out the definition of intellectual content and provided the support platform and funding. All authors read and approved the final manuscript.

## Conflict of Interest

The authors declare that the research was conducted in the absence of any commercial or financial relationships that could be construed as a potential conflict of interest.

## Abbreviations

*S. uberis*: *Streptococcus uberis*
MECs: mammary epithelial cells
Baf-A1: bafilomycin A1
VOTH: VO-ohpic trihydrate
siRNA: small interfering RNA
Rapa: rapamycin
3-MA: 3-methyladenine
LC3: microtubule-related protein 1 light chain 3
p62/SQSTM1: sequestosome-1
ULK1: unc-51-like kinase 1
ATG13: autophagy-related protein 13
ATG5: autophagy related protein 5
PI3K: phosphoinositide 3-kinase
PTEN: phosphatase activity of tensin homolog deleted on chromosome 10
Akt/PKB: protein kinase B
mTOR: mammalian target of rapamycin
4EBP1: eukaryotic initiation factor 4E-binding protein 1
S6K: ribosomal protein S6 kinase
PIP_3_: phosphatidylinositol 3,4,5-trisphosphate
PIP_2_: phosphatidylinositol 4,5-bisphosphate
NAGase: *N*-acetyl-β-D-glucosaminidase
LDH: lactic dehydrogenase
IKK: inhibitor of nuclear factor kappa-B kinase
IκB: inhibitor of nuclear factor kappa-B kinase
NF-κB: nuclear factor kappa B
TEM: transmission electron microscopy.
