# Supplementary material for: Taurine Alleviates Streptococcus uberis-Induced Inflammation by Activating Autophagy in Mammary Epithelial Cells
Source: Front Immunol. 2021 Mar 12;12:631113. doi: 10.3389/fimmu.2021.631113 (PMC7996097; doi:10.3389/fimmu.2021.631113)
Supplement: Supplementary file 1 [file Data_Sheet_1.PDF]

Supplementary Material

Supplementary Figure 1

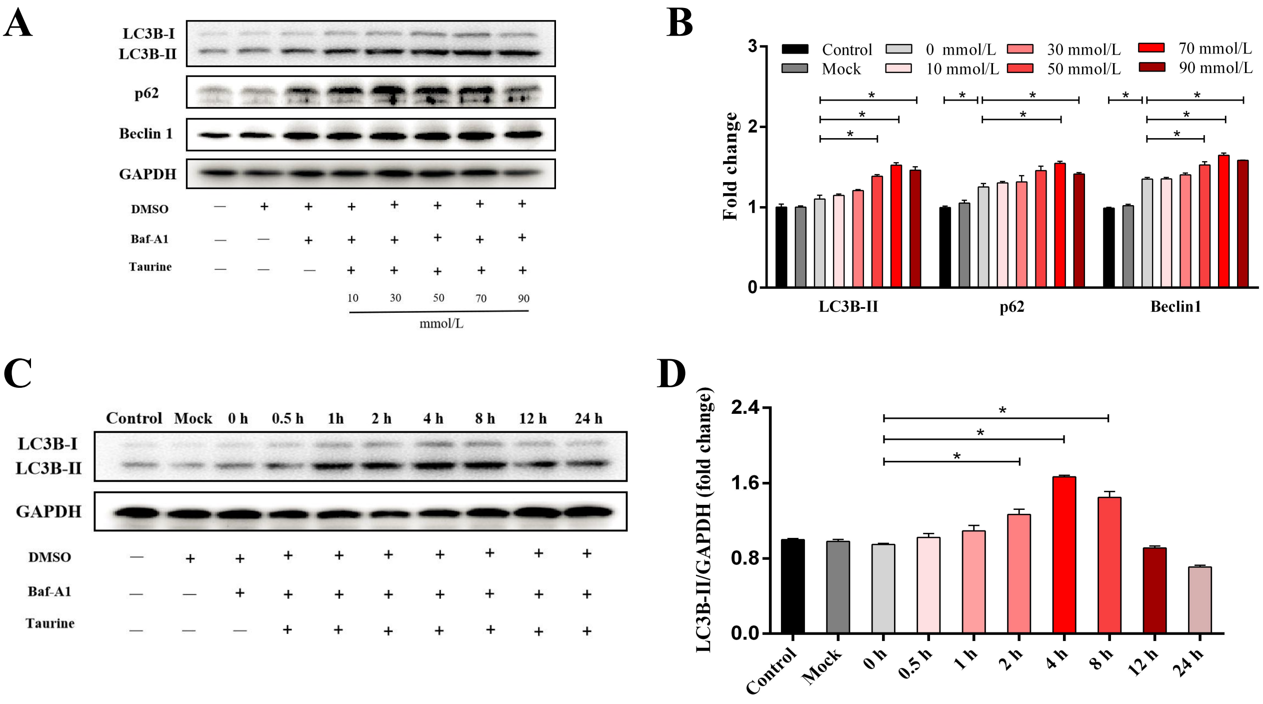

Supplementary Figure 1(A-B) Immunoblots of total protein from MAC-T cells. Some cells were incubated with 50 nmol·L^{-1} Baf-A1 for 1 h before treatment with different taurine concentrations. Quantification of the ratios between LC3B-II, p62, and Beclin 1, as determined by densitometric scanning of immunoblots and normalization to GAPDH. **(C-D)** Immunoblots of total protein from MAC-T cells under different treatments. Some cells were incubated with 50 nmol·L^{-1} Baf-A1 for 1 h before treatment with 70 mmol·L^{-1} taurine for different durations. Data are presented as mean ± SEM. $^{*}P < 0.05$ (significantly different) between the indicated groups; $^{\#}P < 0.05$ (significantly different) between the corresponding negative control groups.

Supplementary Figure 2

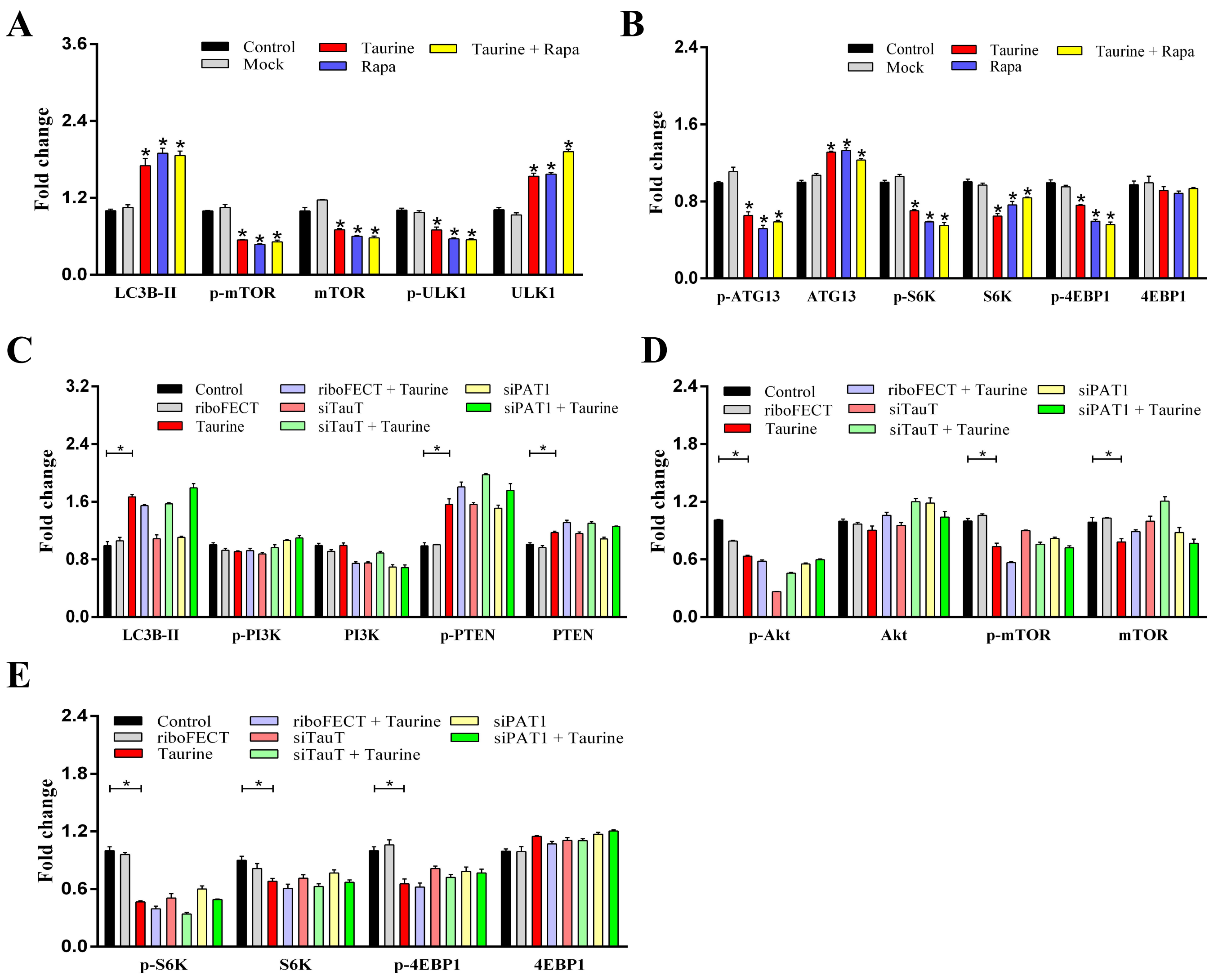

Supplementary Figure 2(A-B) Cells were treated with 70 mmol·L^{-1} taurine for 4 h or 100 nmol·L^{-1} Rapa for 2 h or both; all cells were then probed for the total and phosphorylated (p-) proteins shown. **(C-E)** Representative images of immunoblots of total protein from MAC-T cells transfected with 20 nmol·L^{-1} siNC, siTauT, siPAT 1 for 48 h before treatment with 70 mmol·L^{-1} taurine for 4 h. Quantification of the ratio between total and phosphorylated (p-) proteins, as determined by densitometric scanning of immunoblots and normalization to GAPDH (n=3). Data are presented as mean ± SEM. **P* < 0.05 (significantly different) between the indicated groups; #*P* < 0.05 (significantly different) between the corresponding negative control groups.

Supplementary Figure 3

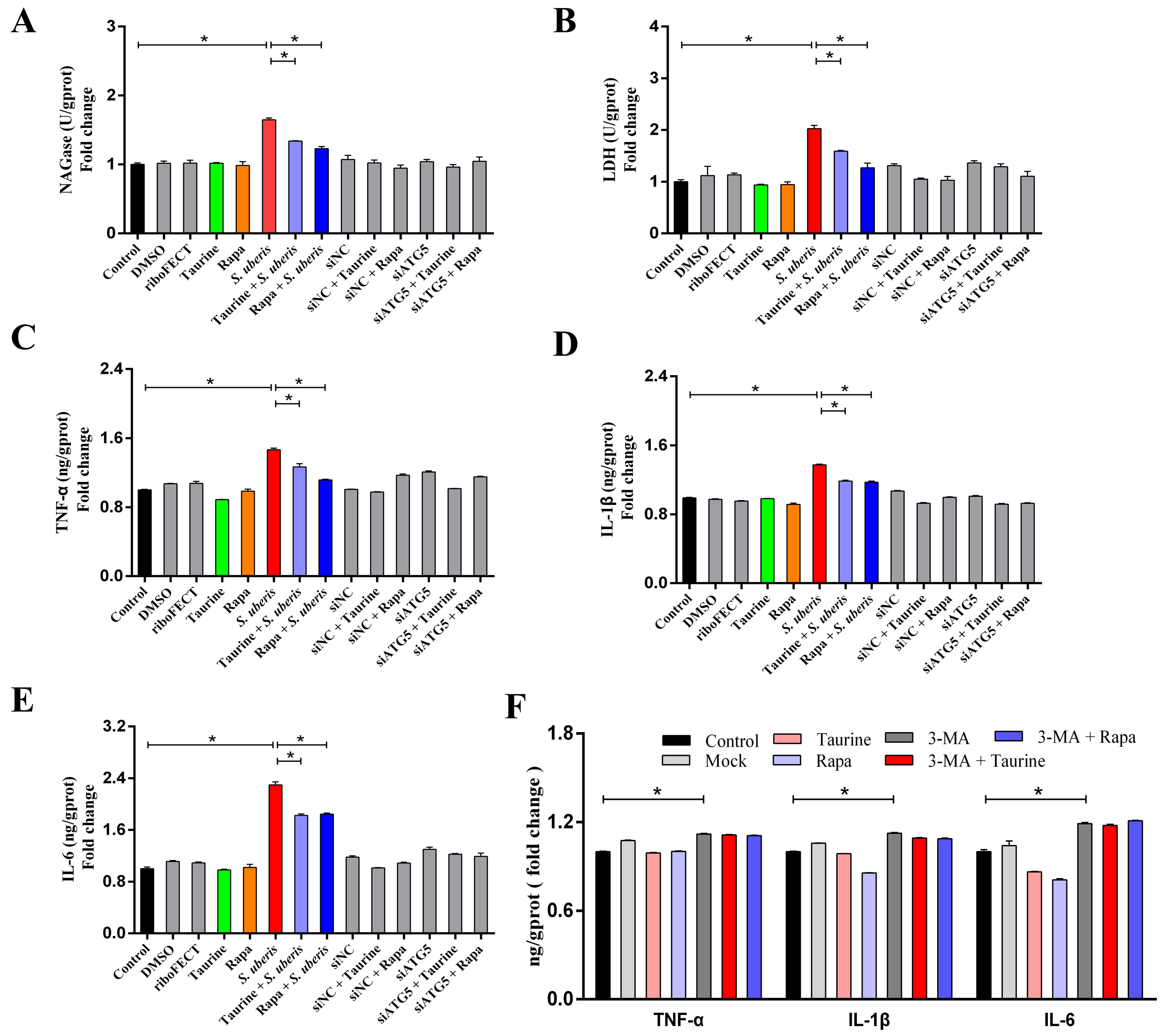

Supplementary Figure 3(A-B) NAGase and LDH activities in supernatant were determined using commercial kits. MAC-T cells transfected with 20 nmol·L^{-1} siNC or siATG5 for 48 h were then treated with 70 mmol·L^{-1} taurine for 4 h or 100 nmol·L^{-1} Rapa for 2 h, and then infected with *S. uberis* in mid-exponential phase at MOI of 10 for 4 h. **(C-E)** TNF-α, IL-1β, and IL-6 concentrations in supernatant were determined by ELISA. MAC-T cells transfected with 20 nmol·L^{-1} siNC or siATG5 for 48 h then treated with 70 mmol·L^{-1} taurine for 4 h or 100 nmol·L^{-1} Rapa for 2 h, and then infected with *S. uberis* in mid-exponential phase at MOI of 10 for 4 h. **(F)** TNF-α, IL-1β, and IL-6 concentrations in supernatant were determined by ELISA. MAC-T cells incubated with 5 mmol·L^{-1} 3-MA for 24 h were then treated with 70 mmol·L^{-1} taurine for 4 h or 100 nmol·L^{-1} Rapa for 2 h. Data are presented as mean ± SEM. *$P < 0.05$ (significantly different) between the indicated groups; #$P < 0.05$ (significantly different) between the corresponding negative control groups.

Supplementary Figure 4

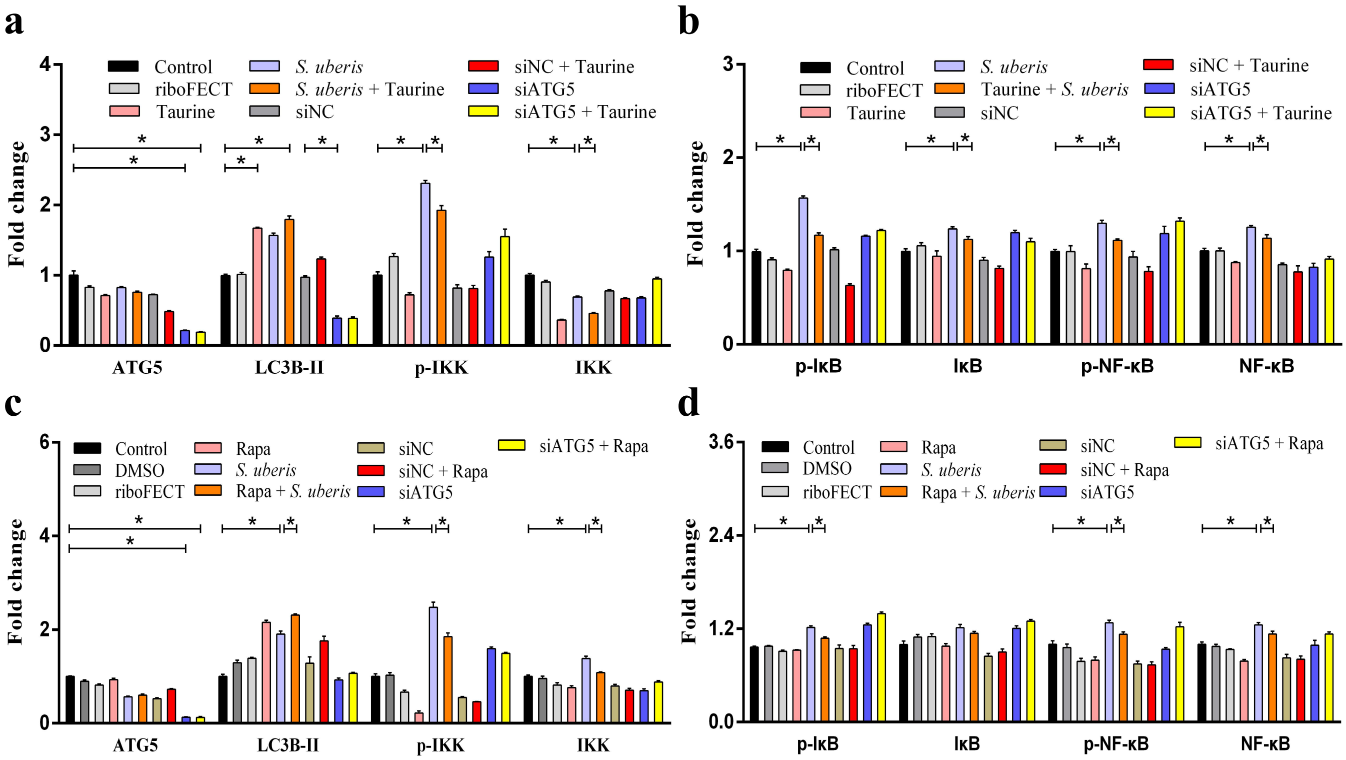

Supplementary Figure 4(A-D) Representative immunoblots of total protein from MAC-T cells transfected with 20 nmol·L^{-1} siNC or siATG5 for 48 h prior to treatment with 70 mmol·L^{-1} taurine for 4 h or 100 nmol·L^{-1} Rapa for 2 h, and then infected with *S. uberis* in mid-exponential phase at MOI of 10 for 4 h. Quantification of the ratio between total and phosphorylated (p-) proteins, as determined by densitometric scanning of immunoblots and normalization to GAPDH (n=3). Data are presented as mean ± SEM. **P* < 0.05 (significantly different) between the indicated groups; #*P* < 0.05 (significantly different) between the corresponding negative control groups.
